# The association of diabetes with risk of prostate cancer defined by clinical and molecular features

**DOI:** 10.1038/s41416-020-0910-y

**Published:** 2020-05-29

**Authors:** Xiaoshuang Feng, Mingyang Song, Mark A. Preston, Wenjie Ma, Yang Hu, Claire H. Pernar, Konrad H. Stopsack, Ericka M. Ebot, Benjamin C. Fu, Yiwen Zhang, Ni Li, Min Dai, Lydia Liu, Edward L. Giovannucci, Lorelei A. Mucci

**Affiliations:** 1 Office of Cancer Screening, National Cancer Center/National Clinical Research Center for Cancer / Cancer Hospital, Chinese Academy of Medical Sciences and Peking Union Medical College, Beijing, China; 2 Department of Epidemiology, Harvard T. H. Chan School of Public Health, Boston, MA USA; 3 Department of Nutrition, Harvard T.H. Chan School of Public Health, Boston, MA USA; 4 Clinical and Translational Epidemiology Unit and Division of Gastroenterology, Massachusetts General Hospital and Harvard Medical School, Boston, MA USA; 5 Division of Urology, Brigham and Women’s Hospital, Boston, MA USA; 6 Department of Medicine, Memorial Sloan Kettering Cancer Center, New York, NY USA; 7 Channing Division of Network Medicine, Brigham and Women’s Hospital and Harvard Medical School, Boston, MA USA

**Keywords:** Cancer prevention, Risk factors, Cancer epidemiology

## Abstract

**Background:**

To prospectively examine the association between diabetes and risk of prostate cancer defined by clinical and molecular features.

**Methods:**

A total of 49,392 men from the Health Professionals Follow-up Study (HPFS) were followed from 1986 to 2014. Data on self-reported diabetes were collected at baseline and updated biennially. Clinical features of prostate cancer included localised, advanced, lethal, low-grade, intermediate-grade, and high-grade. Molecular features included *TMPRSS2: ERG* and *PTEN* subtypes. Cox proportional hazards regression models were used to evaluate the association between diabetes and incidence of subtype-specific prostate cancer.

**Results:**

During 28 years of follow-up, we documented 6733 incident prostate cancer cases. Relative to men free from diabetes, men with diabetes had lower risks of total (HR: 0.82, 95% CI: 0.75–0.90), localised (HR: 0.82, 95% CI: 0.74–0.92), low-and intermediate-grade prostate cancer (HR: 0.77, 95% CI: 0.66–0.90; HR: 0.77, 95% CI: 0.65–0.91, respectively). For molecular subtypes, the HRs for ERG-negative and ERG-positive cases were 0.63 (0.42–0.95) and 0.72 (0.46–1.12); and for PTEN-intact and PTEN-loss cases were 0.69 (0.48–0.98) and 0.52 (0.19–1.41), respectively.

**Conclusion:**

Besides providing advanced evidence for the inverse association between diabetes and prostate cancer, this study is the first to report associations between diabetes and ERG/PTEN defined prostate cancers.

## Background

Prostate cancer and type 2 diabetes mellitus are two of the most common chronic diseases that afflict the aging male population.^1^ The epidemiological findings of consistent inverse associations between diabetes and prostate cancer risk in multiple studies have represented an enigma.^2–5^ Although meta-analyses have reported similar inverse associations for diabetes with aggressive and nonaggressive prostate cancers,^6^ some studies have found a stronger inverse association for nonaggressive cancers, suggesting prostate-specific antigen (PSA) screening history and/or low PSA level among males with diabetes might lead to detection bias and underlie the inverse association.^7,8^

Additionally, it has been suggested that certain molecular subtypes of prostate cancer, including the *TMPRSS2:ERG* fusion and *PTEN* loss,^9^ are associated with biochemical recurrence or worse prognosis, even beyond that predicted by the Gleason score and tumour stage.^10^ Prior studies have reported a close biological relationship between ERG and PTEN,^11,12^ which together may delineate distinct prostate cancer subtypes with different prognosis; for example, relative to PTEN loss and ERG-negative prostate cancer patients, patients with PTEN intact and ERG positive/negative were observed to have better prognosis.^13^ Moreover, our group also found that risk factors associated with energy balance, such as high body mass index (BMI) and low physical activity are specifically associated with risk of *TMPRSS2: ERG* cancers.^14,15^ The association of diabetes with respect to the two molecular subtypes is of biological interest; however, no studies have been investigated to date.

Therefore, we examined the associations between diabetes and risk of developing prostate cancer defined by clinical features (stage, grade, and lethality) and molecular (*TMPRSS2: ERG, PTEN)* subtype taking into account screening patterns. We further examined whether the associations differed by diabetes lifestyle risk factors; and the potential effect of medication use on prostate cancer risk among men with diabetes.

## Methods

### Study population

The Health Professionals Follow-up Study (HPFS) is an ongoing prospective cohort of men initiated in 1986 among 51,529 health professionals of age 40–75 years in the US at baseline. After excluding those who died, reported having cancers (excluding non-melanoma skin cancer) prior to baseline (*n* = 2092), or missing date of birth or prostate cancer diagnosis (*n* = 45), a total of 49,392 men were included in the current study.

### Assessment of diabetes

On the baseline and subsequent follow-up biennial questionnaires, participants were asked if and when they had been diagnosed with diabetes by a physician. To confirm the self-reported cases of physician-diagnosed diabetes, a subsequent mailing was sent for ascertainment to obtain details about the date of diagnosis, symptoms, diagnostic tests and hypoglycaemic treatment. In addition, the information of regular use of insulin or oral hypoglycaemic medications was queried in the questionnaire. Diabetes cases identified before 1998 were defined according to the National Diabetes Data Group criteria,^16^ and the American Diabetes Association criteria was applied after 1998.^17^ The validity of the supplementary questionnaire for diabetes diagnosis has been confirmed in prior studies in HPFS, with 97% accuracy.^18^ Therefore, we took self-reported diabetes as the exposure. Duration of diabetes was calculated by subtracting the date of diagnosis from the date of the most recent completed questionnaire, and categorised as ≤1 year, 1.1–6 years, 6.1–15 years and >15 years.

### Assessment of covariates

Information on age, race and height was collected at baseline; aspirin use, weight and lifestyles were collected at baseline and on each biennial questionnaire; waist circumference was assessed in 1987; family history of prostate cancer in father or brother was collected in 1990; statins use was collected at 1990 and on each subsequent biennial questionnaire. Current BMI and BMI at age 21 were calculated as self-reported weight divided by the square of height reported (kg/m^2^). Information on PSA screening was first asked in 1994 when men were asked to report their most recent PSA test, and in subsequent biennial cycles, they were asked whether they had a PSA test in the past 2 years. Dietary and nutrient intakes were assessed by a validated food frequency questionnaire at baseline and every 4 years thereafter.

### Ascertainment of prostate cancer cases

Incident prostate cancers were initially self-reported on questionnaires, followed by confirming cancer diagnosis and extracting clinical and treatment information through medical records and pathology reports.^19^ Deaths were reported by family members, or identified through the National Death Index, with >98% sensitivity;^20^ Prostate cancer-specific death was determined by review of death certificates and medical records by an endpoint committee of physicians. Archival prostate tumour tissue from about half of HPFS participants diagnosed with prostate cancer was retrieved and undergone central histopathologic reviewed by study pathologists for the standardised tumour grading.

Stage T1a prostate cancer cases (*n* = 295) were excluded from this analysis since these cases are incidentally diagnosed and prone to detection bias. We classified clinical subtypes of prostate cancer as localised (stage T1 or T2 and N0, M0), advanced (stage T3b, T4, N1, or M1), lethal (distant metastases or prostate cancer was the cause of death); low-grade (Gleason 2–6), intermediate-grade (Gleason 7), and high-grade (Gleason 8–10) prostate cancer using information from prostatectomy or biopsy pathology reports.

A total of 5932 prostate cancer cases were accumulated between 1986 and 2009, among 2509 prostate cancer patients who received radical prostatectomy (RP) or transurethral resection of the prostate (TURP), we leveraged tumour ERG and PTEN immunohistochemistry (IHC) data (available for 949 and 757 cases, respectively) from tissue microarrays (TMAs).^21^ Tumours were classified as ERG positive if the case had positive ERG staining within prostate cancer epithelial cells on at least one TMA core. Tumours were classified as PTEN-loss if PTEN immunohistochemistry expression was either markedly decreased or entirely lost across >10% of tumour cells compared with surrounding benign glands or stroma.^13^ Relative to cases without IHC data, cases with IHC data were diagnosed at a more localised stage, had tumours with lower Gleason scores, had lower PSA levels, and were more often diagnosed in earlier years (Supplementary Table 1).

### Statistical analysis

Person-time for participants was calculated from the return of the baseline questionnaire until the date of prostate cancer diagnosis, death, loss to follow-up, or the end of the follow-up (January 2014), whichever came first. For molecular defined prostate cancer outcomes, follow-up ended on 31 December 2009 because this was the last year a case assayed for ERG and PTEN was diagnosed. Consistent with our previous study,^2^ we evaluated the association with status (no vs. yes) and duration of diabetes (≤1, 1.1–6, 6.1–15 and >15 years). Cox proportional hazards regression models were applied to calculate the multivariable hazard ratios (HRs) and 95% confidence intervals (95% CIs). Age in months and calendar year at start of follow-up of each 2-year questionnaire cycle were used as stratification variables in the model. In multivariable analyses, we adjusted for race (white, African American, Asian American, other), family history of prostate cancer in father/brother (yes, no), height (≤68, >68–70, >70–72, >72 inches), BMI at age 21 (<20, 20 to <22.5, 22.5 to <25, ≥25 kg/m^2^), current BMI (<21, 21 to <25, 25 to <30, ≥30 kg/m^2^), smoking (never, former/quit >10 years ago, former/quit ≤10 years ago, current), lagged PSA testing history (yes, no, lagged by one period to avoid counting diagnostic PSA tests), lagged PSA testing in >50% of possible time periods (yes, no, lagged by one period to avoid counting diagnostic PSA tests), physical activity (quintiles of metabolic equivalent of task (MET)-h/week), total calories (quintiles of kcal/day), calcium intake (quintiles of mg/day), tomato sauce intake (quintiles of servings/week), fish intake (quintiles of servings/week), and coffee intake (quintiles of cups/day). All covariates, except race, height, and BMI at age 21, were updated with each questionnaire. Information from the prior questionnaire was carried forward for missing values.

An extension of Cox modelling that allows for exposure associations to vary by disease subtype was applied in the current study,^22,23^ and the details of this competing risks method have been described in our previous study.^24^ In brief, this model allowed for estimating HRs separately for the risk of diagnosis with ERG-positive cancer and ERG-negative cancer versus no cancer, and PTEN-intact and PTEN-loss versus no cancer. We tested heterogeneity across hazard ratios using likelihood ratio tests.^25^ In further, we applied inverse probability weights (IPW) to the competing risk model to validly estimate the association between history of diabetes and prostate cancer incidence by ERG and PTEN expression subtype. The method to create these weights have been described before.^24^ In brief, we first set weights to be 1 for subjects free from cancer and to be zero for patients who developed cancer but did not have RP or TURP tissue, second, we applied weights that accounted for clinical characteristics at and timing of diagnosis for patients who had tissue for IHC assay. To further investigate potential confounding by PSA screening, stratified analyses were applied for PSA screening history (yes, no), PSA screening intensity (>50% and ≤50% of reporting a PSA test in possible time periods) among PSA screened men, and PSA test level (normal, elevated) among PSA screened men.

We also conducted analyses of joint effect of diabetes and its high-risk lifestyle factors, including current BMI (≥30 vs. <30 kg/m^2^), BMI at 21 (≥23 vs. <23 kg/m^2^), waist circumference (≥40 vs. <40 inches), physical activity (<9 vs. ≥9 METS-h/week), and family history of diabetes (yes vs. no), with the risk of prostate cancer. We used a Wald test to examine whether the cross-product terms between these variables and diabetes status were statistically significant. Finally, we restricted to the diabetes population to test associations between use of aspirin, statins, and anti-diabetic medications and prostate risk.

All statistical analyses were conducted using the SAS software (Version 9.4; SAS Institute, Cary, NC, USA). All statistical tests were two-sided, and the significance level was set at *P* < 0.05.

## Results

### Participant characteristics by diabetes status and durations

During 1,078,832 person-years of follow-up, we documented a total of 6733 incident cases of prostate cancer. Among molecular defined prostate cancer cases, 452 (48%) prostate cancers were ERG fusion positive, 109 (14%) prostate cancers were PTEN loss. Midway through follow-up in 2002, 10% of men had reported a history of diabetes. Participants with diabetes were older, more likely to smoke and have a family history of diabetes. Moreover, participants with diabetes were more likely to have higher BMI and waist circumference and were less likely to be physically active. Patients with longer duration of diabetes generally tended to be more likely to have a family history of diabetes, and a higher proportion of aspirin and statins use (Table 1).

**Table 1:** Age-standardised characteristics of participants according to status and duration of type2 diabetes in the Health Professionals Follow-up Study, mid-way through follow-up in 2002.

	Self-report history of diabetes		Duration of diabetes, years			
	No (*n* = 34538)	Yes (*n* = 3753)	≤1 (*n* = 264)	1.1–6 (*n* = 1142)	6.1–15 (*n* = 1393)	>15 (*n* = 954)
Mean age, years^a^	67.8 (9.0)	71.3 (8.8)	68.7 (8.1)	69.4 (9.1)	71.9 (8.5)	73.3 (8.6)
White, %	96	93	96	95	92	91
Family history of prostate cancer, %	12	11	13	11	12	11
Median duration of diabetes, years	–	6.8 (3.1, 14.4)	0.3 (0.1, 0.8)	3.7 (2.4, 4.7)	9.4 (7.6, 11.8)	19.7 (18.2, 32.2)
Family history of diabetes, %	24	48	45	45	47	51
PSA screening in 1994, %	37	37	44	34	39	36
History of PSA test, %	86	90	94	93	89	84
Tested ≥50% of possible time periods, %	64	63	70	64	64	59
Median BMI at age 21 years, kg/m^2^	22.9 (21.2, 24.4)	23.7 (21.5, 25.8)	23.2 (21.1, 25.7)	23.6 (21.5, 25.8)	23.7 (21.8, 26.5)	23.5 (21.3, 25.8)
Median BMI, kg/m^2^	25.7 (23.7, 28.0)	27.5 (24.8, 30.9)	27.7 (24.9, 31.1)	27.8 (25.1, 31.0)	28.1 (25.1, 31.6)	26.4 (23.8, 29.3)
Median waist, inches	36.5 (34.8, 39.0)	39.3 (37.0, 42.5)	39.0 (37.0, 41.0)	39.5 (37.0, 42.3)	40.3 (37.8, 43.8)	38.0 (35.0, 40.8)
Median height, inches	70.0 (68.0,72.0)	70.0 (68.0,72.0)	70.0 (68.0,72.0)	70.0 (68.0,72.0)	70.0 (68.0,72.0)	70.0 (68.0,72.0)
Current smoker, %	5	5	6	5	6	5
Median total physical activity, MET-h/week	22.6 (11.8, 37.9)	16.2 (7.7, 29.1)	17.8 (8.6, 34.7)	16.3 (8.2, 28.3)	15.0 (7.1, 27.2)	18.1 (7.9, 30.9)
Median total energy intake, kcal/day	1921 (1595, 2316)	1896 (1559, 2280)	1914 (1559, 2274)	1908 (1566, 2291)	1893 (1550, 2275)	1889 (1578, 2332)
Median tomato sauce intake, servings/week	0.8 (0.5,1.4)	0.8 (0.5,1.4)	0.9 (0.5,1.6)	0.8 (0.5,1.4)	0.8 (0.5,1.4)	0.7 (0.5,1.3)
Median coffee intake, cups/day	1.4 (0.5,2.5)	1.4 (0.6,2.4)	1.5 (0.6,2.4)	1.4 (0.6,2.3)	1.4 (0.5,2.4)	1.4 (0.6,2.5)
Median fish intake servings/week	1.5 (1.0,3.0)	1.5 (1.0,3.0)	2.0 (1.0,3.0)	1.5 (1.0,3.0)	1.5 (1.0,3.0)	1.5 (1.0,3.0)
Median calcium intake, mg/day	882 (712,1127)	887 (718,1141)	884 (705,1142)	876 (717,1132)	864 (701,1112)	938 (766,1197)
Use of aspirin, %	40	45	46	46	42	47
Use of statins, %	22	36	42	35	36	35

Values are means (SD) or medians (Q25, Q75) for continuous variables; variables are standardised to the age distribution of the study population.

Values of polytomous variables may not sum to 100% due to rounding.

*BMI* body mass index, *PSA* prostate-specific antigen, *MET* metabolic equivalent of task.

^a^Value is not age adjusted.

### Diabetes and risk of prostate cancer, by clinical and pathologic tumour characteristics

Table 2 shows that history of diabetes is inversely associated with prostate cancer risk (HR: 0.82, 95% CI: 0.75–0.90), particularly in localised (HR: 0.82, 95% CI: 0.74–0.92) and low-and intermediate-grade prostate cancer (HR: 0.77, 95% CI: 0.66–0.90; HR: 0.77, 95% CI: 0.65–0.91, respectively). For advanced (HR: 0.83, 95% CI: 0.61–1.14) and lethal (HR: 0.86, 95% CI: 0.68–1.08), the reduced risks were also observed but not statistically significant. Meanwhile, the magnitude of this association was stronger in men whose duration of diabetes was longer than 1 year (*P*_trend_ = 0.0067); compared to men without diabetes history, the HRs (95% CI) for ≤ 1 year, 1.1–6 years, 6.1–15 years and >15 years duration of diabetes groups, for total prostate cancer were 1.23 (0.92–1.64), 0.79 (0.67–0.93), 0.83 (0.72–0.96) and 0.77 (0.64–0.92), respectively. Notably, a positive association with low-grade prostate cancer was observed for men with ≤1-year duration of diabetes (HR: 1.61, 95% CI: 1.07–2.42).

**Table 2:** Hazard ratios and 95% confidence intervals for clinical featured prostate cancer risk among men by status and duration of diabetes in the Health Professionals Follow-up Study, 1986–2014.

	Non-diabetic	Diabetics	≤1 year	1.1–6 years	6.1–15 years	>15 years	*P*_trend_^c^
Person-years	996731	82101	5074	24482	30113	22432	
Total prostate cancer							
No. incident cases	6210	523	47	145	201	130	
HR^a^ (95% CI)	1.00 (ref)	0.80 (0.73–0.88)	1.21 (0.91–1.62)	0.77 (0.65–0.91)	0.81 (0.70–0.93)	0.74 (0.62–0.88)	0.0064
HR^b^ (95% CI)	1.00 (ref)	0.82 (0.75–0.90)	1.23 (0.92–1.64)	0.79 (0.67–0.93)	0.83 (0.72–0.96)	0.77 (0.64–0.92)	0.0067
Localised prostate cancer							
No. incident cases	4533	381	36	96	155	94	
HR^a^ (95% CI)	1.00 (ref)	0.79 (0.71–0.88)	1.26 (0.90–1.75)	0.69 (0.56–0.85)	0.83 (0.71–0.97)	0.75 (0.61–0.92)	0.13
HR^b^ (95% CI)	1.00 (ref)	0.82 (0.74–0.92)	1.28 (0.92–1.78)	0.71 (0.58–0.87)	0.86 (0.73–1.01)	0.78 (0.64–0.96)	0.091
Advanced prostate cancer							
No. incident cases	572	44	2	16	12	14	
HR^a^ (95% CI)	1.00 (ref)	0.80 (0.59–1.09)	0.60 (0.15–2.43)	1.02 (0.62–1.69)	0.63 (0.35–1.12)	0.83 (0.48–1.42)	0.32
HR^b^ (95% CI)	1.00 (ref)	0.83 (0.61–1.14)	0.63 (0.15–2.54)	1.04 (0.63–1.74)	0.65 (0.37–1.17)	0.87 (0.51–1.50)	0.035
Lethal prostate cancer							
No. incident cases	922	79	6	23	21	29	
HR^a^ (95% CI)	1.00 (ref)	0.86 (0.68–1.09)	1.20 (0.53–2.70)	0.90 (0.59–1.37)	0.63 (0.41–0.97)	1.06 (0.73–1.54)	0.30
HR^b^ (95% CI)	1.00 (ref)	0.86 (0.68–1.08)	1.19 (0.53–2.67)	0.89 (0.58–1.35)	0.62 (0.40–0.97)	1.05 (0.72–1.53)	0.31
Low-grade prostate cancer							
No. incident cases	2483	186	24	47	71	44	
HR^a^ (95% CI)	1.00 (ref)	0.73 (0.63–0.85)	1.54 (1.02–2.31)	0.63 (0.47–0.84)	0.72 (0.57–0.92)	0.67 (0.50–0.91)	0.030
HR^b^ (95% CI)	1.00 (ref)	0.77 (0.66–0.90)	1.61 (1.07–2.42)	0.66 (0.49–0.89)	0.76 (0.59–0.96)	0.71 (0.53–0.96)	0.031
Intermediate-grade prostate cancer							
No. incident cases	1981	150	15	42	59	34	
HR^a^ (95% CI)	1.00 (ref)	0.72 (0.61–0.86)	1.21 (0.72–2.02)	0.70 (0.51–0.95)	0.73 (0.56–0.95)	0.63 (0.45–0.89)	0.087
HR^b^ (95% CI)	1.00 (ref)	0.77 (0.65–0.91)	1.26 (0.75–2.10)	0.72 (0.53–0.99)	0.78 (0.60–1.02)	0.68 (0.48–0.95)	0.12
High-grade prostate cancer							
No. incident cases	811	85	2	24	32	27	
HR^a^ (95% CI)	1.00 (ref)	0.95 (0.75–1.19)	0.40 (0.10–1.60)	0.97 (0.65–1.47)	0.94 (0.65–1.34)	1.04 (0.70–1.53)	0.68
HR^b^ (95% CI)	1.00 (ref)	0.94 (0.75–1.18)	0.40 (0.10–1.59)	0.96 (0.64–1.45)	0.92 (0.64–1.32)	1.06 (0.71–1.56)	0.84

Localised cases: stage T1 or T2 and N0, M0; advanced cases: stage T3b, T4, N1, or M1; lethal cases: distant metastases or prostate cancer was the cause of death

Low-grade cases: Gleason 2–6; intermediate-grade cases: Gleason 7; high-grade cases: Gleason 8–10.

*CI* confidence interval, *HR* hazard ratio, *PSA* prostate-specific antigen.

^a^Adjusted for age and calendar time.

^b^Adjusted for age, calendar time, race, family history of prostate cancer in father or brother, height, body mass index at current and age 21 years, smoking, lagged PSA testing history, lagged PSA testing in >50% of possible time periods, physical activity, total calories, calcium intake, tomato sauce intake, fish intake, and coffee intake.

^c^Value for *P* trend among men with diabetes.

### Diabetes and risk of prostate cancer, by molecular tumour characteristics

In Table 3, the HRs for diabetes and ERG-negative and ERG-positive prostate cancers were 0.63 (0.42–0.95) and 0.72 (0.46–1.12); and for PTEN-intact and PTEN-loss prostate cancer were 0.69 (0.48–0.98) and 0.52 (0.19–1.41), respectively. However, no significant interaction was observed between diabetes and ERG or PTEN subtypes (*P*-heterogeneity = 0.67 and 0.59, respectively).

**Table 3:** Hazard ratios and 95% confidence intervals for molecular featured prostate cancer risk among men with or without diabetes in the Health Professionals Follow-up Study, 1986–2009.

	Non-diabetic	Diabetics	*P*_heterogeneity_
By ERG status			
ERG-positive prostate cancer			
No. incident cases	431	21	
HR^a^ (95% CI)	1.00 (ref)	0.64 (0.41–1.00)	0.70
HR^b^ (95% CI)	1.00 (ref)	0.72 (0.46–1.12)	0.67
ERG-negative prostate cancer			
No. incident cases	473	24	
HR^a^ (95% CI)	1.00 (ref)	0.57 (0.38–0.86)	
HR^b^ (95% CI)	1.00 (ref)	0.63 (0.42–0.95)	
By PTEN status			
PTEN-intact prostate cancer			
No. incident cases	616	32	
HR^a^ (95% CI)	1.00 (ref)	0.62 (0.44–0.88)	0.58
HR^b^ (95% CI)	1.00 (ref)	0.69 (0.48–0.98)	0.59
PTEN-loss prostate cancer			
No. incident cases	105	4	
HR^a^ (95% CI)	1.00 (ref)	0.46 (0.17–1.27)	
HR^b^ (95% *CI*)	1.00 (ref)	0.52 (0.19–1.41)	

*CI* confidence interval, *HR* hazard ratio, *PSA* prostate-specific antigen.

^a^Adjusted for age and calendar time.

^b^Adjusted for age, calendar time, race, family history of prostate cancer in father or brother, height, body mass index at current and age 21 years, smoking, lagged PSA testing history, lagged PSA testing in >50% of possible time periods, physical activity, total calories, calcium intake, tomato sauce intake, fish intake, and coffee intake.

### Stratified analysis for diabetes and risk of prostate cancer, by PSA screening history

Table 4 presents the stratified analysis by PSA screening history. We found similar associations between diabetes and prostate cancer risk among men who received regular PSA screening (HR: 0.83, 95% CI: 0.74–0.92) or not (HR: 0.81, 95% CI: 0.67–0.98). In addition, we found an inverse association among PSA screened men with high intensity of screening (>50% of reporting a PSA test in possible time periods) (HR: 0.80, 95% CI: 0.71–0.90), and among screened men who had a normal PSA test value (HR: 0.85, 95% CI: 0.74–0.99). Similar inverse associations were observed for the risk of less aggressive prostate cancers (localised, or low-/intermediate-grade) but not for aggressive cases (advanced, lethal or high-grade).

**Table 4:** Hazard ratios and 95% confidence intervals for stratified analysis according to PSA test history for the association between diabetes and prostate cancer risk.

	Total prostate cancer		Localized, or low-/intermediate-grade prostate cancer		Advanced, lethal or high-grade prostate cancer	
	Non-diabetic	Diabetics	Non-diabetic	Diabetics	Non-diabetic	Diabetics
PSA test history						
No						
No. incident cases	1973	123	1511	86	305	22
HR^a^ (95% CI)	1.00 (ref)	0.81 (0.67–0.98)	1.00 (ref)	0.75 (0.60–0.94)	1.00 (ref)	0.97 (0.62–1.52)
Yes						
No. incident cases	4237	400	3665	331	284	34
HR^a^ (95% CI)	1.00 (ref)	0.83 (0.74–0.92)	1.00 (ref)	0.81 (0.72–0.91)	1.00 (ref)	0.96 (0.66–1.38)
PSA test intensity among PSA screened men						
<50%						
No. incident cases	909	88	747	71	83	10
HR^a^ (95% CI)	1.00 (ref)	0.91 (0.72–1.15)	1.00 (ref)	0.93 (0.72–1.20)	1.00 (ref)	1.04 (0.47–2.29)
≥50%						
No. incident cases	3328	312	2918	260	201	24
HR^a^ (95% CI)	1.00 (ref)	0.80 (0.71–0.90)	1.00 (ref)	0.78 (0.69–0.89)	1.00 (ref)	0.90 (0.58–1.40)
PSA level among PSA screened men^b^						
Normal						
No. incident cases	2220	211	1932	169	145	22
HR^a^ (95% CI)	1.00 (ref)	0.85 (0.74–0.99)	1.00 (ref)	0.80 (0.68–0.94)	1.00 (ref)	1.30 (0.81–2.09)
Elevated						
No. incident cases	1493	137	1303	117	88	7
HR^a^ (95% CI)	1.00 (ref)	0.98 (0.81–1.19)	1.00 (ref)	1.00 (0.81–1.23)	1.00 (ref)	0.62 (0.24–1.61)

Localised cases: stage T1 or T2 and N0, M0; advanced cases: stage T3b, T4, N1, or M1; lethal cases: distant metastases or prostate cancer was the cause of death.

Low-grade cases: Gleason 2–6; intermediate-grade cases: Gleason 7; high-grade cases: Gleason 8–10.

*CI* confidence interval, *HR* hazard ratio, *PSA* prostate-specific antigen.

^a^Adjusted for age, calendar time, race, family history of prostate cancer in father or brother, height, body mass index at current and age 21 years, smoking, physical activity, total calories, calcium intake, tomato sauce intake, fish intake and coffee intake.

^b^Study period was 1994–2014.

### Diabetes and its lifestyle risk factors with risk of prostate cancer

The independent and joint associations of current high BMI (≥30 kg/m^2^), BMI at 21 (≥23 kg/m^2^), waist circumference (≥40 inches), physical activity (≥9 METS-h/week), family history of diabetes, and diabetes are showed in Table 5. In particular, there was a stronger inverse association between diabetes and risk of prostate cancer among those with high waist circumference, although no significant interaction was observed (*P*-interaction = 0.22).

**Table 5:** Analyses of joint effect of diabetes and lifestyles with the risk of prostate cancer in the Health Professionals Follow-up Study, 1986–2014.

Subgroup		Cases	Person-years	HR^a^ (95% CI)	*P*_interaction_
Current BMI	Diabetes				0.53
<30 kg/m^2^	No	5040	793650	1.00 (ref)	
≥30 kg/m^2^	No	601	99611	1.00 (0.91–1.09)	
<30 kg/m^2^	Yes	364	55369	0.83 (0.75–0.93)	
≥30 kg/m^2^	Yes	106	18641	0.76 (0.63–0.93)	
BMI at 21	Diabetes				0.92
<23 kg/m^2^	No	3502	523249	1.00 (ref)	
≥23 kg/m^2^	No	2708	473482	0.95 (0.90–1.00)	
<23 kg/m^2^	Yes	268	39463	0.83 (0.73–0.94)	
≥23 kg/m^2^	Yes	255	42638	0.78 (0.68–0.89)	
Waist circumference	Diabetes				0.22
<40 inches	No	3516	519597	1.00 (ref)	
≥40 inches	No	908	131738	0.93 (0.86–1.02)	
<40 inches	Yes	201	28895	0.83 (0.71–0.95)	
≥40 inches	Yes	130	21937	0.66 (0.55–0.80)	
Physical activity	Diabetes				0.67
<9 METS-h/week	No	1187	232989	1.00 (ref)	
≥9 METS-h/week	No	5023	763741	1.04 (0.97–1.11)	
<9 METS-h/week	Yes	137	23859	0.80 (0.67–0.95)	
≥9 METS-h/week	Yes	386	58243	0.86 (0.77–0.97)	
Family history of diabetes	Diabetes				0.67
No	No	4739	752316	1.00 (ref)	
Yes	No	1471	244415	0.95 (0.89–1.00)	
No	Yes	277	42784	0.85 (0.75–0.97)	
Yes	Yes	246	39317	0.77 (0.68–0.88)	

*CI* confidence interval, *HR* hazard ratio, *BMI* body mass index, *PSA* prostate-specific antigen.

^a^Adjusted for age, calendar time, race, family history of prostate cancer in father or brother, height, body mass index at current and age 21 years, smoking, lagged PSA testing history, lagged PSA testing in >50% of possible time periods, physical activity, total calories, calcium intake, tomato sauce intake, fish intake, and coffee intake. Of note, variables examined in this Table were not adjusted for.

### Medications and risk of prostate cancer among patients with diabetes

In addition, we tested that whether use of oral anti-diabetic medications, insulin, aspirin, and statins affect prostate cancer risk among men with diabetes (Table 6). Results showed that there were no statistically significant associations (HR: 0.80, 95% CI: 0.58–1.12; HR: 0.96, 95% CI: 0.52–1.77; HR: 1.14, 95% CI: 0.94–1.39; HR: 1.14, 95% CI: 0.91–1.43, respectively).

**Table 6:** Medications use and risk of prostate cancer, in men with diabetes.

Medication use	Cases	Person-year	HR^a^ (95% CI)
Oral anti-diabetic medication^b^			
No	147	21506	1.00 (ref)
Yes	94	14909	0.80 (0.58–1.12)
Insulin^b^			
No	223	34359	1.00 (ref)
Yes	18	2056	0.96 (0.52–1.77)
Insulin or oral anti-diabetic medication^b^			
No	138	20427	1.00 (ref)
Yes	103	15987	0.82 (0.59–1.14)
Aspirin			
No	250	44345	1.00 (ref)
Yes	273	37756	1.14 (0.94–1.39)
Statins^c^			
No	317	48252	1.00 (ref)
Yes	189	27445	1.14 (0.91–1.43)

*CI* confidence interval, *HR* hazard ratio, *PSA* prostate-specific antigen.

^a^Adjusted for age, calendar time, race, family history of prostate cancer in father or brother, height, body mass index at current and age 21 years, smoking, lagged PSA testing history, lagged PSA testing in >50% of possible time periods, physical activity, total calories, calcium intake, tomato sauce intake, fish intake, and coffee intake.

^b^Males without missing information of anti-diabetic medication.

^c^Study period was 1990–2014.

## Discussion

In this large, updated analysis within the HPFS cohort with up to 28 years of follow-up, we confirmed inverse associations in the risk of prostate cancer among men with long-term diabetes. The associations were particularly strong for localised and low-/intermediate-grade prostate cancer. Of note, the inverse associations remained even when controlling for PSA screening history and frequency. Additionally, for the first time, we present data on the association between diabetes and risk of prostate cancer based on two molecular subtypes.

Twenty years ago, findings from the HPFS were the first prospective data with more than one thousand incident prostate cancer cases to demonstrate a statistically significant inverse association between diabetes and risk of prostate cancer.^26^ This finding has since been replicated in several cohort studies among different populations, including the 2009 analysis in HPFS.^2^ However, the inverse association differed by disease aggressiveness,^3,7,8^ and was primarily observed in the localised, low-grade prostate cancer, which keeps in line with our updated results. Considering the aggressiveness of prostate cancer is defined based on subsequent outcomes after diagnosis, such as metastasis and death, we assumed that diabetes may not be inversely associated with the most clinically relevant outcomes of prostate cancer, and a meta-analysis study showed that pre-existing type-2 diabetes is non-significantly positively associated with prostate cancer-specific mortality (RR: 1.17, 95% CI: 0.96–1.42) in prostate cancer patients.^27^

Although a previous meta-analysis found no statistically significant departure from linearity between length of time being diabetic and prostate cancer risk (*p* < 0.34),^28^ we observed the trend of linear association when four groups of diabetes duration were analysed as continuous variable. And the inverse associations were more frequently observed in men with longer duration of diabetes;^5,8,29–31^ even a positive relation could be observed in some studies for the shorter diabetes duration.^5,8,29,31^

One possible mechanism to explain the inverse association between diabetes and prostate cancer is the relative insulin-deficient environment in long-term diabetes, resulting in lower plasma insulin (C-peptide) and insulin-like growth factor-1 (IGF-1) levels in diabetics compared to non-diabetics.^32^ This is important given consistent findings in prospective studies that higher circulating levels of IGF-1 are associated with an increased risk of prostate cancer, particularly for the non-aggressive and low-grade disease.^33^ Additionally, circulating levels of the insulin-like growth factor-binding protein 2 (IGFBP2) have been positively correlated with insulin sensitivity over prolonged periods,^34^ and the risk of developing diabetes was 5-fold lower for IGFBP2 levels in the top quintile versus the lowest quintile;^35^ however, IGFBP2 concentration was positively associated with prostate cancer risk.^33^ Another potential mechanism is the genetic link, several loci, especially hepatocyte nuclear factor-1 β gene (HNF1β), have been reported to be associated with the risk of both diabetes and prostate cancer;^36,37^ however, mediation analyses provided insufficient evidence for the inverse relationship between diabetes and prostate cancer risk is mediated through diabetes risk variants.^38,39^ Our previous prospective study indicated that ERG positive tumours were characterised by higher expression of insulin receptor and IGF-1 receptor, compared with ERG-negative tumours.^40^ In addition, experimental studies found that PTEN mutations may reduce the risk of type 2 diabetes owing to enhanced insulin sensitivity.^41^ Although our findings of inverse associations between diabetes and ERG-negative and PTEN-intact disease aligned with the hypothesis above, given the analyses by ERG and PTEN status used a smaller number of cases than analyses of prostate cancer overall, chance might have played a role in the different results across diabetes status, and the results need to be confirmed by larger studies.

Given the stronger inverse associations for diabetes with less aggressive prostate cancer, there are lingering concerns that PSA screening could lead to detection bias for the relation. First, prior studies indicated that the participation rate for PSA test could be higher^8,42,43^ or lower^44^ for men with diabetes compared to men without. Second, in men without cancer, PSA levels in diabetics are lower than in men without diabetes, which could contribute to reduced detection rates of prostate cancer, particularly the localised.

To address these two issues, similar with the study conducted in Israel^8^ and our cohort,^14^ we first adjusted for lagged PSA testing and intensity in the main analysis, and we additionally undertook several stratified analyses. The inverse association for diabetes remained in the subgroup of men with regular PSA testing, which were consistent with our previous results^2^ and findings from the Prostate, Lung, Colorectal, and Ovarian (PLCO) Screening Trial cohort.^7^ Moreover, when stratified on men with normal PSA levels at testing, the reduced risk of overall and nonaggressive prostate cancer among males with diabetes persisted. Therefore, similar with previous studies considering PSA level and screening frequency,^3^ our results suggested that detection bias might contribute to part of the inverse association but is unlikely to fully explain the link between diabetes and prostate cancer. Studies have consistently reported an inverse association between obesity and the risk of less aggressive prostate cancer.^45,46^ Obesity has broad systemic effects including lower circulating testosterone levels.^47^ The slightly stronger inverse associations between diabetes and prostate cancer among obese males, especially those with central obesity, and males with low physical activity level suggests a potential modified effect of obesity on the association. Meanwhile, the slightly stronger association has also been observed in males with a family history of diabetes, and a nationwide study from Sweden has reported that family history of type 2 diabetes mellitus was associated with a lower incidence of prostate cancer, and the risk was even lower for those with more than one affected relatives.^48^ The potential mechanisms may be attributed to the genetic factors or shared familial factors, such as obesity.

Medications such as aspirin and statins have been recommended to be used in patients with diabetes for the prevention of cardiovascular events;^49^ together with anti-diabetic medications, they have shown a decreased risk of prostate cancer.^50^ Data from this prospective study showed that there may be no association between these medications and overall prostate cancer risk in men with diabetes. Although there may be misclassification due to self-report, this is expected to be nondifferential in HPFS, where medical professionals repeatedly reported on medication use before cancer diagnosis.

There are several potential strengths and limitations in our study to consider in interpreting the findings. First, we relied on self-reported diabetes, which may lead to the misclassification of exposure. However, the cohort is comprised of male health professionals, and we have shown the accuracy of self-reported cases with physician-diagnosed diabetes in our cohort was very high (97%). Second, our results might be influenced by detection bias. However, the detailed information available on PSA testing history allowed us adjusted and stratified the potential confounding by PSA test in our results. Moreover, the molecular subtype of prostate cancer may be less susceptible to screening and detection biases, which offered stronger evidence for the association between diabetes and prostate cancer. Third, the ERG and PTEN-featured prostate cancer cases were derived from males who received RP or TURP, but when the inverse probability weighting method was used to balance the potential bias, the results were similar with the unweighted analysis (Supplementary Table 2). The strengths of our study include the prospective study design, the high follow-up rates on questionnaires, with >90% follow-up in each cycle,^51^ and the 28 years of follow-up for cancer incidence and mortality, which enabled us to examine the association between long-term diabetes and different clinical featured prostate cancers with considerable statistical power. Moreover, we have detailed covariate data to control for potential confounding and undertook the sub-analysis to assess potential for bias.

In summary, the updated results from this large prospective male cohort provided converging evidence for the inverse association between diabetes and prostate cancer, particularly for the nonaggressive prostate cancer, suggesting that the presence of diabetes may influence the frequency and interpretation of screening tests for prostate cancer. In addition, this is the first study to our knowledge to report the association between diabetes and molecular defined prostate cancer, which might contribute to the interpretation of the inverse association between diabetes and prostate cancer and may help researchers to follow-up with potential mechanisms underlying the association for future targets of intervention.

## Supplementary information

supplementary table 1 and table 2

## Data Availability

The datasets generated during and/or analysed during the current study are not publicly available due to confidentiality reasons, but anonymised versions may be available from the corresponding author on reasonable request.
