# Supplementary material for: The association of diabetes with risk of prostate cancer defined by clinical and molecular features
Source: Br J Cancer. 2020 May 29;123(4):657–65. doi: 10.1038/s41416-020-0910-y (PMC7435261; doi:10.1038/s41416-020-0910-y)
Supplement: Supplementary file 1 — supplementary table 1 and table 2 [file 41416_2020_910_MOESM1_ESM.docx]

**Supplementary Table 1. Characteristics of participants with prostate cancer at the time of diagnosis, by treated and IHC status, Health Professionals Follow-up Study, 1986-2009**

|  | **Participants treated with RP or TURP** | | |  | **Participants treated otherwise IHC data unavailable** |
| --- | --- | --- | --- | --- | --- |
|  | **ERG data available** | **PTEN data available** | **ERG or PTEN data unavailable** |  |  |
| Number | 949 | 757 | 1541 |  | 3423 |
| Mean Age at diagnosis*, years | 65.7 (6.0) | 65.7 (6.0) | 66.1 (6.1) |  | 73.0 (6.9) |
| Year of diagnosis |  |  |  |  |  |
| 1986-1990 | 9.5 | 8.2 | 9.5 |  | 8.5 |
| 1991-1995 | 33.3 | 29.3 | 26.7 |  | 22.8 |
| 1996-2000 | 29.8 | 27.6 | 24.4 |  | 25.9 |
| 2001-2005 | 19.3 | 24.4 | 21.7 |  | 27.5 |
| 2006-2009 | 8.1 | 10.6 | 17.7 |  | 15.3 |
| Median PSA Level at Diagnosis, ng/ml | 6.8 (4.8,10.6) | 6.6 (4.9,10.3) | 5.9 (4.4,8.8) |  | 7.3 (5.1,12.0) |
| Clinical Stage, % |  |  |  |  |  |
| T1, T2 | 92.9 | 93.7 | 90.5 |  | 70.8 |
| T3, | 3.3 | 3.0 | 2.5 |  | 3.3 |
| T4, N1, M1 | 2.1 | 1.9 | 2.1 |  | 6.9 |
| Unknown | 1.7 | 1.4 | 4.8 |  | 19.0 |
| Clinical Gleason Score, % |  |  |  |  |  |
| <7 | 59.1 | 57.5 | 61.0 |  | 44.1 |
| 7 | 30.7 | 31.3 | 25.5 |  | 20.2 |
| >7 | 10.1 | 11.0 | 7.0 |  | 10.4 |
| Unknown | 0.1 | 0.1 | 6.5 |  | 25.2 |

IHC = immunohistochemistry; RP = radical prostatectomy; TURP = transurethral resection of the prostate; PSA = prostate-specific antigen.

Values are means (SD) or medians (Q25, Q75) for continuous variables; variables are standardized to the age distribution of the study population.

Values of polytomous variables may not sum to 100% due to rounding.

* Value is not age adjusted.

| **Supplementary table 2. Hazard ratios and 95% confidence intervals for molecular featured prostate cancer risk among men with or without diabetes,** **using inverse probability weighting, Health Professionals Follow-up Study, 1986-2009** | | | |
| --- | --- | --- | --- |
|  | **Non-diabetic** | **Diabetics** | ***P _heterogeneity_*** |
| **By ERG status** |  |  |  |
| **ERG-positive prostate cancer** |  |  |  |
| No. incident cases | 431 | 21 |  |
| HR ^a^ (95% *CI*) | 1.00 (ref) | 0.61 (0.39-0.98) | 0.56 |
| HR ^b^ (95% *CI*) | 1.00 (ref) | 0.70 (0.44-1.12) | 0.60 |
| **ERG-negative prostate cancer** |  |  |  |
| No. incident cases | 473 | 24 |  |
| HR ^a^ (95% *CI*) | 1.00 (ref) | 0.51 (0.32-0.81) |  |
| HR ^b^ (95% *CI*) | 1.00 (ref) | 0.57 (0.36-0.91) |  |
| **By PTEN status** |  |  |  |
| **PTEN-intact prostate cancer** |  |  |  |
| No. incident cases | 616 | 32 |  |
| HR ^a^ (95% *CI*) | 1.00 (ref) | 0.59 (0.40-0.87) | 0.47 |
| HR ^b^ (95% *CI*) | 1.00 (ref) | 0.65 (0.44-0.95) | 0.58 |
| **PTEN-loss prostate cancer** |  |  |  |
| No. incident cases | 105 | 4 |  |
| HR ^a^ (95% *CI*) | 1.00 (ref) | 0.37 (0.12-1.22) |  |
| HR ^b^ (95% *CI*) | 1.00 (ref) | 0.46 (0.14-1.50) |  |

CI = confidence interval; HR = hazard ratio; PSA = prostate-specific antigen.

a: Adjusted for age and calendar time.

b: Adjusted for age, calendar time, race, family history of prostate cancer in father or brother, height, body mass index at current and age 21 years, smoking, lagged PSA testing history, lagged PSA testing in >50% of possible time periods, physical activity, total calories, calcium intake, tomato sauce intake, fish intake, and coffee intake.
